# Music and Noise: Same or Different? What Our Body Tells Us

**DOI:** 10.3389/fpsyg.2019.01153

**Published:** 2019-06-25

**Authors:** Mark Reybrouck, Piotr Podlipniak, David Welch

**Affiliations:** ^1^ Musicology Research Group, Faculty of Arts, KU Leuven-University of Leuven, Leuven, Belgium; ^2^ IPEM, Department of Art History, Musicology and Theatre Studies, Ghent, Belgium; ^3^ Institute of Musicology, Adam Mickiewicz University in Poznań, Poznań, Poland; ^4^ Audiology Section, School of Population Health, University of Auckland, Auckland, New Zealand

**Keywords:** hearing damage, leisure noise, noise as biological stressor, prelethal use of sound, biomarkers of loud music listening, loud music, noise annoyance

## Abstract

In this article, we consider music and noise in terms of vibrational and transferable energy as well as from the evolutionary significance of the hearing system of *Homo sapiens*. Music and sound impinge upon our body and our mind and we can react to both either positively or negatively. Much depends, in this regard, on the frequency spectrum and the level of the sound stimuli, which may sometimes make it possible to set music apart from noise. There are, however, two levels of description: the physical-acoustic description of the sound and the subjective-psychological reactions by the listeners. Starting from a vibrational approach to sound and music, we first investigate how sound may activate the sense of touch and the vestibular system of the inner ear besides the sense of hearing. We then touch upon distinct issues such as the relation between low-frequency sounds and annoyance, the harmful effect of loud sound and noise, the direct effects of overstimulation with sound, the indirect effects of unwanted sounds as related to auditory neurology, and the widespread phenomenon of liking loud sound and music, both from the point of view of behavioral and psychological aspects.

## Introduction

Music, as an informationally rich or “thick” event, cannot be reduced to perceptual dimensions such as pitch, rhythms, etc. or to its physical constituents. What really matters, on the contrary, is the dynamic, multifaceted, and multisensorial phenomenon of the music (Eidsheim, 2015, p. 2) with effects that can be “devastating, physically brutal, mysterious, erotic, moving, boring, pleasing, enervating, or uncomfortable, generally embarrassing, subjective, and resistant to the gnostic” (Abbate, 2004, p. 514). This means that our actual involvement with music is mainly “experienced” rather than being solely “reasoned” and “interpreted” (Reybrouck, 2014, 2017; Reybrouck and Eerola, 2017): it is *drastic* rather than *gnostic* to use Jankélévitch’s terms (Jankélévitch, 2003). Music’s ontological status, in this view, should be changed from an external, knowable object to an unfolding phenomenon that arises through complex material interactions of human physiology with the sounds (Eidsheim, 2015, p. 2). The musical experience, then, can be described in terms of a specific relationship between the material bodies of the listener and the vibrational properties of the sounds at one level while also containing in it a more conscious appreciation of the traditional musical forms and parameters.

On the other hand, music may be considered just as sound that impinges on us *via* our sensory apparatus and our interpretations of the signals we receive. Music and by extension all sounds, in this view, are considered in vibrational terms as *transferable energy*, which impinges upon our body and our senses (Eidsheim, 2015, p. 16). From the perspective of acoustics, music is energy that pulsates through and across a medium; the structural interpretation and esthetic conceptualization of it occur when the sound has been processed, decoded, and interpreted in our nervous system. This vibrational energy, moreover, is not restricted to the sense of hearing: it activates not only the auditory system but also the sense of touch (e.g., Huang et al., 2012) and the vestibular system of the inner ear (Todd, 1993, 2001; Todd and Cody, 2000). The vestibular system emerges early in both phylogeny and ontogeny (Trainor et al., 2009) and interacts with the auditory system, both at the subcortical level (Oertel and Young, 2004) and at the cortical level (Phillips-Silver and Trainor, 2007, 2008). At the phylogenetic level, it was the first sensory system to develop in evolution and ontogenetically the first to develop in the womb (Romand, 1992). This may be interpreted to suggest that a sense of orientation and acceleration is more fundamental to perception than vision and hearing. As such, the experience of music involves the simultaneous activation of multiple sensory modalities. Furthermore, the preconscious responses continue even once sound is processed by our sensory systems by activating the autonomic nervous system, which controls physiological functions such as respiration, heart function, digestion, the hormonal system, and the immune system (Maschke et al., 2000; Maschke, 2004).

This power to influence us on a fundamental level can been observed in the use of sonic weapons to dominate and confuse targets and to destroy the subjectivity of prisoners in the interrogation room (Volcler, 2013), and listening to loud music merely for pleasure. The latter has been described by Cusick as “a shared experience of being touched-without-being-touched by the vibrating air” from which she drew “a deeply sensual, erotic (though not explicitly sexual) feeling of communion with the friends and strangers around me.” And further: “[The experience] was enhanced by the adrenalin rush, the raised blood pressure and heart rate, the ringing that would last for hours in my bones that were the best-known, immediate physical effects of loud music” (Cusick, 2006, p. 6).

## Music and Noise

It has been hypothesized that the esthetic quality of music concerns the balance of sound along several dimensions such as frequency, space, and time (Brattico et al., 2017). Musical information, in this view, is balanced, so that listeners hear meaningful musical information in a distinct way. Noise, on the contrary, is less constrained and is often largely uncontrolled. As such, it has a lot of negative connotations, which refer to both acoustic descriptions and subjective valuations. The term “noise,” moreover, has three main usages: (1) sounds at potentially deafening levels, (2) unwanted sounds, and (3) statistical processes where events are random and uncorrelated, and which give rise to sounds where the waveform follows such a statistical distribution (Keizer, 2010; Hainge, 2013, p. 120). These usages are often confounded in daily use. A good example is in the recent history of the twentieth century Western music. To quote Hainge: “From Schoenberg to Stravinsky to Russolo to Cage to Hendrix to Merzbow, atonality, dissonance, explosions, coughs, splutters, feedback, distortion, glitch, and various shades of noise have done their best to (dis)colour music and to make of it what we had thought it was not” (Hainge, 2013, p. 2). This refers mostly to noise in the third sense, but the second sense (unwantedness) is also invoked. In other words, the delimitation of the concept of music and its currently accepted esthetic canon may be seen as having developed beyond a state of balance to include the uncontrolled and even the unwanted. As such, there has been a broadening of the scope of music, both with respect to the use of the frequency spectrum and its dynamic range. Where traditional “musical sounds” could be commonly located within the optimal zone of stimulation, there is actually a development to accept a shift in the extremes of the frequency spectrum and the loudness levels that are considered as being acceptable.

## Music and the Human Hearing Range

**Figure 1:**
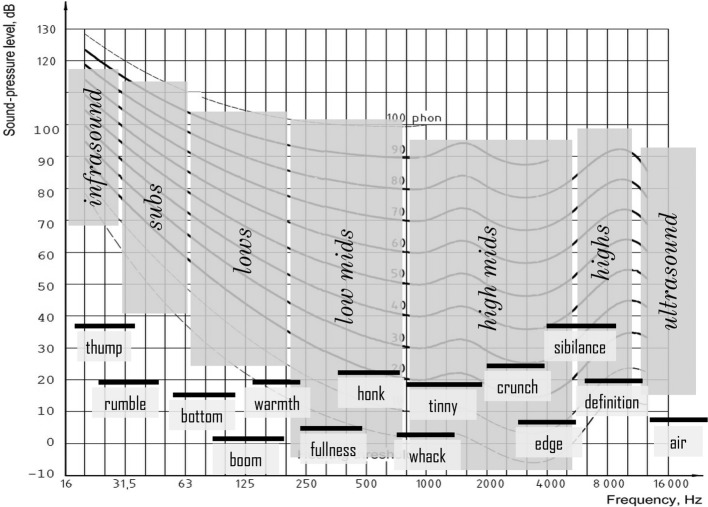
Divisions of the audible spectrum (parsing and feel) plotted onto the equal loudness curves. (Figure reproduced without any changes from Fink, 2018, p. 95 with permission of Oxford Publishing Limited through PLSclear, Ref No: 13971).

In the frequency domain, normal human ears have been supposed to be sensitive for frequencies between 20 and 20,000 Hz, with the highest sensitivity in the frequency range used for processing speech signals (200 Hz to 5,000 Hz). Nonetheless, hearing extends beyond this, and all detectable sounds can be parsed into musical “frequency zones” together with their “feels” (Figure 1) alongside the equal loudness curves (Fletcher and Munson, 1933; Fink, 2018, p. 95).

Recent findings about the sensitivity of the human ear above this range, however, challenge these constraints (Møller and Pedersen, 2004; Fukushima et al., 2014). It has been found that brain electrical activity and regional cerebral blood flow (rCBF) could be demonstrated in a listening experiment with listeners being exposed to gamelan music of Bali, which is extremely rich in high-frequency components. This music provides a particular example of a sound source with two major components, namely a (classically “audible”) low-frequency component (LFC) below 22 kHz and a high-frequency component (HFC) above 22 kHz. Listeners were not able to recognize the HFC when presented in isolation, but the alpha frequency range of their spontaneous electroencephalogram (alpha-EEG), recorded from the occipital region, showed a significant increase when exposed to sound that contained both HFCs and LFCs. This response has been termed the *hypersonic effect* (Oohashi et al., 2000; Fukushima et al., 2014; Ito et al., 2016; Kuribayashi and Nittono, 2017). As such, there have been attempts to explore the psychological effects of such “inaudible” HFCs by using digital audio formats with higher sampling rates (96 kHz), but these studies have not yet convincingly explained the biological mechanism that underlies this phenomenon (Reiss, 2016).

The dividing line between infrasound and low-frequency sound is also open to interpretation. Below 20 Hz, the tonal sensation disappears, with the sound becoming discontinuous in character, changing into a feeling of pressure and turning into a sensation of separate puffs, which can be counted at the level of the single cycles of the sound wave. It is difficult, however, to determine whether sensations at this low frequency level are of a pressure, or tactile, or of an auditory nature (Yeowart et al., 1967; Møller and Pedersen, 2004).

Measurements for hearing thresholds have been made for frequencies of 4 Hz in an acoustic chamber (Watanabe and Møller, 1990) and even for frequencies down to 1.5 Hz with earphone listening (Yeowart et al., 1967; Yeowart, 1976). Much higher stimulus levels, however, are needed below 20 Hz in order to provoke an auditory sensation. It can be postulated, in this regard, that there is a hierarchy of receptors, with the auditory system figuring as the most sensitive, except at the lower frequencies where other receptors may also come into prominence. Examples are the many vibration and contact detectors, which reside in the skin and other organs and which cover different frequency ranges, e.g., the Pacinian corpuscles that are sensitive to threshold displacements of about 0.002 mm at 200 Hz (Johnson, 2001). At lower levels, however, their sensitivity reduces by approximately 50 dB for every 10 Hz. It is unlikely, therefore, that inaudible sound waves would excite these subcutaneous receptors at normal loudness levels (Leventhall, 2007).

## Low-Frequency Sounds, Music, and Annoyance

Sources of infrasound and low-frequency noise can be found in natural phenomena (wind, turbulence, storms, and earthquakes) and man-made sources, such as industrial installations and low-speed machinery (compressors, boilers, ventilation systems, trucks, cars, and ships) as well as a lot of contemporary music, which has been described as adhering to the so-called *bass-culture* with a particular stress on the sheer acoustic materiality of the sub-bass register, ranging from about 20 to 60 Hz (Fink, 2018). Low-frequency noise, moreover, has features that are different from noises at higher frequencies. Many of them are reducible to its extremely pervasive character: it is hardly attenuated by walls and other structures; it can rattle walls and objects; it masks higher frequencies; it crosses great distances with little energy loss; ear protection devices are less effective against it; it is able to produce resonance in the human body; and it causes great subjective reactions (Berglund et al., 1994, 1996).

It is difficult to predict the loudness and annoyance of such low-frequency sound, particularly if measured with dB(A). Although the A filter provides a useful approximation for annoyingness in mid- to high-frequency stationary noise, it underestimates annoyance and perceived loudness for the low-frequency components. Noise that contains high levels of low-frequency noise is perceived as more annoying than higher frequency noise, even at low levels. Comparison between broadband noises centered at 80, 250, 500, and 1,000 Hz showed that the 80-Hz frequency band was more annoying than the other noise bands at equal A-weighted levels (Persson and Björkman, 1988).

Loudness levels alone, however, cannot predict annoyance (Broner, 1978). It has been suggested, in fact, that the type—especially the slope and turnover point of the noise spectra—rather than the loudness level of the low frequency noises is responsible for the feeling of annoyance (Bryan, 1976). At the subjective level, moreover, there are many subjective factors, which influence noise annoyance. A very important aspect is the exposed person’s attitude toward the source as well as the controllability of the stressor (Kuwano et al., 1991; Job, 1993; Berglund et al., 1996).

**Figure 2:**
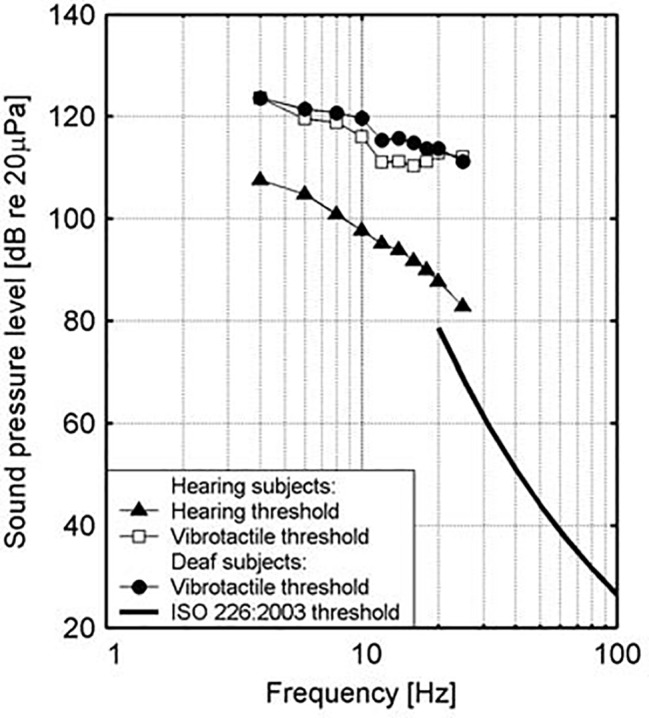
Hearing and vibration thresholds as measured for hearing and deaf subjects. (Figure republished with permission of Sage Publications Ltd. from Landström et al., 1983, permission conveyed through Copyright Clearance Center, Inc., Licence number: 4587301207878).

At a physical level, low frequency noise frequently appears together with tangible vibrations. Sound in air can activate vibrations in housing structures, and low frequency sounds can also arise as a result of vibrations in such structures with room resonance functioning as a possible intensifier of low frequency sound (Maschke, 2004). Similarly, high levels of low-frequency noise can excite vibrations in the human body, particularly the chest region, which resonates in the range of 50–80 Hz (Leventhall, 2007); there is also a 30–40-Hz resonant frequency response for the forehead and face and a 80–90-Hz frequency response for the back of the skull (Takahashi et al., 2002a,b). Intensity levels in excess of normal thresholds, moreover, have been found to be perceived through the body in deaf people. Vibrotactile stimuli may evoke strong responses in the auditory cortex in congenitally deaf persons, which points in the direction of crossmodal plasticity of some areas (supratemporal auditory cortex) of the cortex in the sense that an enhanced ability to detect sudden tactile changes probably seems to compensate for the missing audition in signaling (Levänen et al., 1998; Levänen and Hamdorf, 2001). The extent to which such sensory substitution also holds for normally hearing people, however, is still a matter of debate since the domain of vibrotactile perception is not yet well understood in the context of music perception (Egloff et al., 2011). An older study by Landström et al. (1983) deserves special attention in this regard. It measured hearing and vibrotactile thresholds for normally hearing and deaf subjects and found that vibrotactile thresholds were very similar for both groups, but that this additional way of sensation, which may be possibly connected to vibration, occurs only at levels that are 20–25 dB above the hearing threshold (see Figure 2). At this level of sound pressure level, it is possible to feel vibrations in various parts of the body, such as the bottom, thigh, and calf regions, and a feeling of pressure can be felt in the upper part of the chest and the throat region (Møller and Pedersen, 2004). It is critical, in this regard, to also consider the role of sound wave detection through skeletal bones, the ear, tactile senses, and resonance in body organs (Berglund et al., 1996). It should be noticed, further, that especially in bass culture music with its celebration of the low frequencies, it is held that listening exceeds mere audition by activating the sonic conjunction with amodal perception, in the sense that the bass is not just heard but also felt (Goodman, 2009, p. 236).

## Possible Harmful Effects of Loud Sounds and Noise

The acoustic environment of our industrialized societies has seen a proliferation of noise production across the full range of frequencies during the last decades, as exemplified in the term “noise pollution.” Noise, in fact, can act as a *non-specific biologic stressor* that is able to elicit reactions that prepare the body for fight or flight (Ising and Kruppa, 2004; Babisch, 2005). This brings us to the evolutionary significance of the hearing system of *Homo sapiens*, which may have evolved not only to detect the environment and to function as a *warning system* against possible dangers to ensure survival but also to exchange information between individuals. This takes place by processing the sounds and simultaneously reacting to aspects of the sound itself (e.g., the sound level) while comparing them to categories that are either inherited or previously learned patterns. This determines whether a sound is experienced as something negative (and thus potentially being regarded as annoying noise) or as a normal, acceptable component of the environment (Rylander, 2004). A distinction should be made, further, between “direct” and “indirect” effects of sounds. This holds in particular for loud sounds and noises.

Exposure to noise with sufficient intensity and duration can alter the psychological and physical state, with demonstrable auditory and non-auditory effects on human beings (Basner et al., 2014). There is a difference, however between noise-induced hearing loss, which can be measured for prolonged exposures above some critical levels and annoyance, which may occur at any level.

As such, there has been a bulk of studies on the effects of high levels of noise and sounds on physical and mental health, with respect to both hearing loss and reduced well-being. It is not yet totally clear, however, to what extent this applies equally to high levels of exposure during occupational conditions and workplace activities or during non-work and/or leisure activities, such as music listening (Williams et al., 2010). Care should yet be taken not to generalize too much, as noise is not necessarily harmful. Most real-world noises, e.g., occupy a rather wide frequency spectrum, and there are multiple instances of nature sound such as waterfalls, mountain rivers, rain in the woods, the blowing of the wind, the sound of the surf, and many others, which are considered as “nature’s white noises,” and which may be valued for their relaxing and calming effects (Gould van Praag et al., 2017). Much depends here on the frequency distribution and the relative intensities of the respective frequency bands as well as the functional significance of the perceived sound. But still more important is the attitude toward the source of the noise and personal noise sensitivity. Some people seem predisposed to get upset by noise, and their health is also more impacted in that case (Welch et al., 2013, 2018).

Low-frequency noise and vibrations, further, can also be detrimental to some extent. After some earlier reports on possible harmful effects of low-frequency vibrations (Gavreau et al., 1966; Gavreau, 1968), efforts have been made to discover whether such harmful effects do actually occur. These effects have been described extensively by the research group around Castelo Branco (Castelo Branco et al., 2002; Alves-Pereira and Castelo Branco, 2007), who coined the term *vibroacoustic disease* (VAD) to document a number of symptoms that have been found in people that were exposed to occupational noise. VAD-related lesions seem to be responses of biological tissue to low-frequency noise and have been described in several organs with reported lesions in the nervous system, heart, blood vessels, lymphatics, and respiratory tissues, together with tissue reorganization and neo-formation (da Fonseca et al., 2006; Alves-Pereira and Castelo Branco, 2007). As such, fibrosis has been found in tissues—especially connective tissue—and organs exposed to large pressure amplitude low frequency noise in the absence of visible signs of inflammation, which points into the direction of a protective response of the tissue by increasing the production of elements with a structural role and viscoelastic properties, so as to resist the impact of strong mechanical stress (Oliveira et al., 2013). The reported data were gathered from a whole range of people such as airplane technicians, commercial and military pilots, mechanical engineers, restaurant workers, and disc jockeys, as well as populations that were exposed to low frequency noise as part of their everyday environments (Maschke, 2004). These early reports, however, have been received rather critically and with a lot of skepticism, but a lot of subsequent studies have provided a growing body of evidence to confirm that noise pollution in general may have temporary and permanent effects on humans (and other mammals) (Mayor, 2018).

### Direct Effects of Overstimulation With Sound

The damaging potential of excessive exposure to acoustic stimuli is huge and multifaceted. Several physical effects after acute and chronic exposure to loud sounds have been found, such as cochlear pathology (hair cell loss, spiral ganglion cell apoptosis, and cochlear nerve degeneration), damage to connective tissue, cardiovascular deterioration, and a whole list of symptoms that are grouped under the term “vibroacoustic disease,” embracing mild or severe lesions in the nervous system, heart, blood vessel, lymphatics, and respiratory tissues (Castelo Branco et al., 2002; da Fonseca et al., 2006). A distinction should be made, however, between merely induced aural pain and hearing loss or hearing impairment.

*Aural pain* arises from displacement of the middle ear system beyond its normal operational limits, mainly at low-frequency and infrasound stimulus levels at about 165 dB at 2 Hz, 140–145 dB around 20 Hz, and increasing to about 162 dB. Static pressure produces pain at 175–180 dB, and rupture of the eardrum has been reported at about 185–190 dB (von Gierke and Nixon, 1976; Broner, 1978; Leventhall, 2007). *Hearing impairment*, on the other hand, goes beyond the mere sensation of pain and can be clinically assessed as an increase in the threshold of hearing, either as a temporary (TTS) or a permanent threshold shift (PTS). There is wide agreement that exposure to sound levels below 70 dBA does not produce hearing damage, regardless of the duration of exposure. Exposure for more than 8 h per day to sound levels in excess of 85 dBA Leq, on the contrary, is potentially hazardous over years of exposure, with damage being dependent on sound pressure and time of exposure in terms of hours per day as well as the number of years for which a person is so exposed. The major causes of hearing loss are occupational exposure (workplace), community noise, recreational noise (listening to loud music), as well as a variety of other causes, such as trauma, ototoxic drugs, infection, and heredity. Given the widespread use of electronically amplified music for long periods *via* smartphones, noise-induced hearing loss (NIHL) has been observed in younger people (NIH, 1990; Bulla, 2003; Goines and Hagler, 2007). Thus, NIHL, both temporary and permanent, is a recognized effect of excessive and prolonged exposure to music.

**Figure 3:**
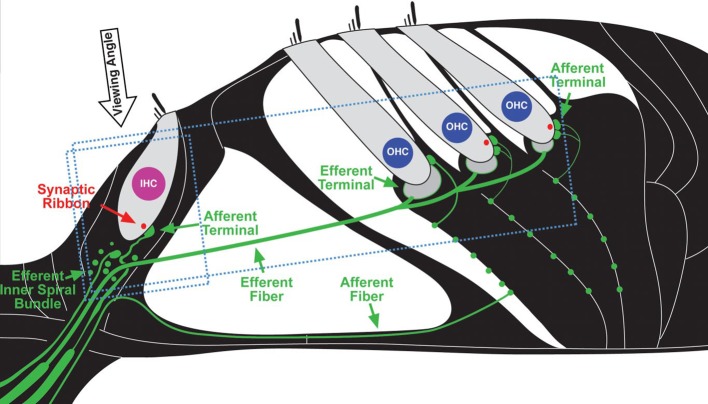
Schematic depiction of cochlear sensory epithelium showing inner (IHC) and outer hair cells (OHC) and their afferent innervation as they appear in neurofilament (green) and synaptic ribbon protein (red). (Figure republished without any changes with permission of Society of Neuroscience from Kujawa and Liberman, 2009, p. 14080; permission conveyed through Copyright Clearance Center, Inc., Licence number: 4587290719756).

**Figure 4:**
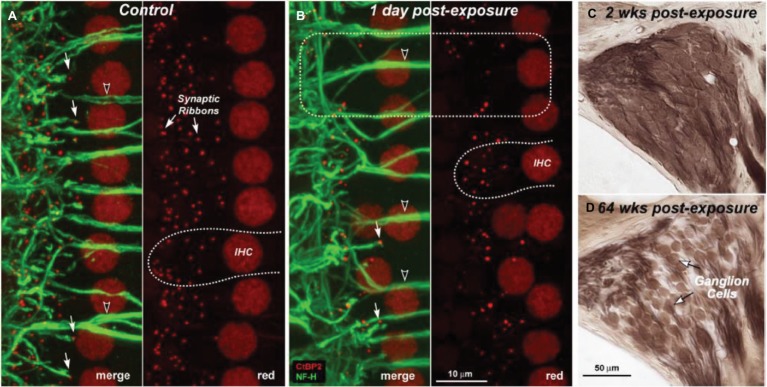
Depiction of loss of cochlear synaptic terminals **(B)** and delayed loss of cochlear ganglion cells **(C,D)** as compared to normal cochlea **(A)**. Figure A and B show synaptic ribbons (red) and cochlear nerve dendrites (green) in the inner hair cell area of a control **(A)** and an exposed ear **(B)**. Merged images show juxtaposed presynaptic ribbons and postsynaptic terminals in both control and exposed ears (**A,B**: filled arrows), and the lack of both in denervated regions (**B**: dashed box). Cochlear sections show normal density of ganglion cells 2 weeks post-exposure **(C)** compared with diffuse loss after 64 weeks **(D)**. (Figure republished without any changes with permission of Society of Neuroscience from Kujawa and Liberman, 2009, p. 14080; permission conveyed through Copyright Clearance Center, Inc., Licence number: 4587290719756).

The problem of *NIHL* is a major one. Exposures that damage hearing are not necessarily painful or annoying. After overexposure, moreover, the hearing loss may apparently recover (temporary threshold shift) or stabilize at an elevated level (permanent threshold shift; Liberman and Dodds, 1984; Clark, 1991; Ryan et al., 2016), though apparent recovery may reflect inadequacy in the hearing testing techniques available. There are, in fact, many sites of noise-induced damage in the ear. One is the destruction of cochlear hair cells or damage to their mechano-sensory hair bundles. Hair cells transduce sound-evoked mechanical motion into receptor potentials, which, in turn, lead to neurotransmitter releases at their synapses with cochlear afferent fibers (see Figures 3, 4). Damage of these cells is visible quite soon after overexposure, and hair cell death can continue for days (Wang et al., 2002). Another site is the synapses and the auditory nerve itself, especially the fibers that respond in high sound-level environments. In animals, damage and loss of these cells may be delayed by months and may progress for years, which make it difficult to diagnose. To the best of our knowledge, this has not yet been done in humans, though the types of damage that occur are consistent with the so-called “hidden hearing loss,” which means that there is normal hearing when measured in quiet conditions alongside the inability to identify speech in a noisy background (Kujawa and Liberman, 2006, 2009; Perez and Bao, 2011).

### Indirect Effects of Unwanted Sound: Auditory Neurology

**Figure 5:**
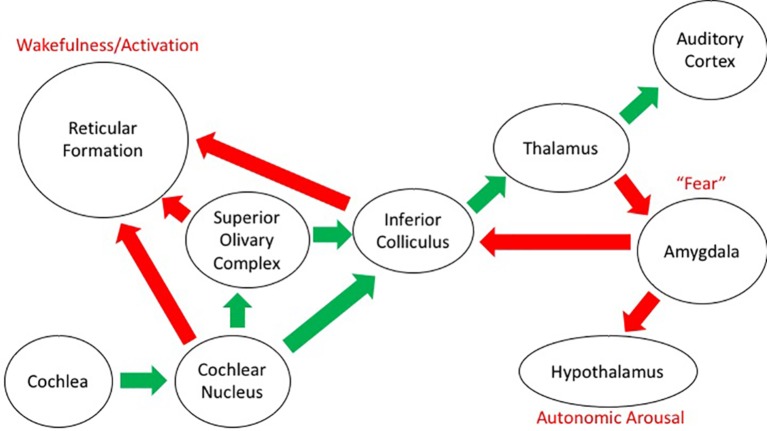
Schematic diagram of the auditory projections in the brain. The “classical” auditory pathway conveying information about sound from the ear to the cortex is shown by the green arrows. The other projections from the auditory system to structures relating to emotion and arousal are shown with the red arrows. General descriptors of function in these structures are included in red text.

Sound impinges on our body in a direct and indirect way. Besides the need to analyze the sound itself in the search for meaning and correspondence with memories of sounds that have proved to be meaningful at earlier occurrences, there is a simultaneous effect on the physiological responses of our body. This is the case, mostly, when a loud sound gets our attention, giving rise to a reaction that combines sound detection with emotional appraisal (Beckerman, 2014). There are, in fact, two kinds of auditory pathway in the central nervous system: besides the classical pathways from the inner ear to the auditory cortex, there are also pathways to the reticular activating system, which has connections with the limbic system and the autonomic nervous system. The pituitary adrenal neuroendocrine system, in particular, is involved in the secretion of corticosteroids, which are involved in the management of stress through the sympathetic-adrenal system that controls the secretion of catecholamine, adrenaline, and noradrenaline (see Figure 5; Koch and Schnitzler, 1997; Kraus and Canlon, 2012; Welch and Fremaux, 2017b).

As such, there are connections with brain centers that control physiological, emotional, and behavioral responses and that affect alertness, mental states, and motor performance. This happens mostly below the level of consciousness and deliberate control and is quite resistant to habituation (Rylander, 2004). Though the general mechanisms behind these reactions are known to some extent (Kraus and Canlon, 2012), there is a large variation in between individuals. As such, it is assumed that genetic factors, previous experience, and the presence of environmental stimuli may play a major role in the sensitivity for noise. It has been also found that differences in 5-HT_1A_ serotonin receptors are related to awareness of the environment and the reception of sound-mediated information (Borg et al., 2003).

There is, further, a distinction to be made between acute reactions to noise exposure and possible lasting effects. *Acute reactions* comprise three major effects: an orienting response, a startle reflex, and a defense/flight reaction, all of them being warning or alert reflexes. The startle responses, in particular, are quite important. They can be defined as a simple defensive response to a sudden acoustic, tactile, or visual stimulus, which may signal proximal threat (Landis and Hunt, 1939; Gelfand, 2009; Parker et al., 2011). The neural circuitry of this response and its primary modulating factor have been described in detail already quite early in the research literature (Davis et al., 1982). In animals, this response is typically measured by the magnitude of whole-body movement; in humans, the most common response measurements are contraction of the orbicularis oculi muscle, cardiac acceleration, and scalp electroencephalographic potential (Blumenthal et al., 2005). *Lasting effects* of stress-induced disturbance of homeostatic equilibrium, on the contrary, are related to imbalance of the autonomous nervous system (ANS), with one branch dominating over the other. This holds in particular for the sympathetic branch being hyperactive and the parasympathetic branch being hypoactive over an extended duration. Energy demands on the system then become excessive and ultimately cannot be met, which results in various pathological conditions, which are labeled as ANS dysfunction, embracing complex and heterogenous disorders and diseases, quite often in conjunction with neurodegenerative diseases, neurodevelopmental disorders, autoimmune diseases, mental disorders, and ischemic stroke or myocardial infarction (Ellis and Thayer, 2010).

## Liking Loud Music: Psychological and Behavioral Aspects

Loud sound has proved to have a lot of impact on our physical and mental health, having both auditory and non-auditory effects (Basner et al., 2014). It is present, moreover, in many activities, which are considered to be recreational, such as fitness centers, sports events, personal audio systems, live music events, bars, and night clubs, which all have high levels of sound (Welch and Fremaux, 2017a,b). Clubbers experience on average an equivalent continuous noise level of 98 dBA Leq over an average attendance time of 5 h a week (Williams et al., 2010). It can be questioned in this regard why people enjoy stimuli that cause discomfort and negative impact on their health. Two aspects should be distinguished in this context: the way listeners experience these loud stimuli and the way they are imposed on them. The latter points to bar managers and DJs who use loud music not only to retain customers, to control the crowd, and even to reduce conflict (Forsyth, 2009) but also for business reasons. Loud sounds stimulate people to drink more due to the higher arousal level and the reduction of social interaction (Guéguen et al., 2004). These motivations should be considered carefully by bar managers as a significant number of young adults consider these sound levels as being too high (Mercier and Hohmann, 2002; Gilles et al., 2013, 2014; Johnson et al., 2014; Beach and Gilliver, 2019). It makes sense, therefore, to delve into the attitude of young adults toward loud music in order to understand this music consumption from a theoretical perspective. Music, in fact, is not merely reducible to the structure of the sound. More important is the psychological impact of sound, which is perceived as a multisensory experience. Music, in this view, is “felt” as well as “heard,” and it can be studied from the point of view of its aural, tactile, or motor induction qualities.

### Bass Culture and Sound as Power

People often like “hot” sound with great penetrating power. This is the celebration of the bass culture with a conception of “sound as power.” It brings us to the widely established attitudes toward loud music as established in particular in adolescents (Landälv et al., 2013). Given that sounds in bars and dance clubs may reach levels in excess of 120 dB SPL and that such loud music is considered to be pleasurable to some, with the loudness itself being a source of pleasure, it can be questioned which attributes of the sound contribute to this experience of pleasure (Todd and Cody, 2000). Loud music, in contrast to industrial noise which mostly has a rather flat broadband frequency character, and which is known to be harmful, is not considered stressful to some up to sound levels of 105 dB Leq. This is exemplified in what is known as the *rock and roll threshold* of around 96 dB Leq, provided that sufficient low frequency energy is present (Dibble, 1995). Live performance frequency spectra, moreover, should require at least a critical difference (from 10 to 30 dB) between the midband energy level and that of the low-frequency band (50–100 Hz), which seems to suggest that part of the source of pleasure is the predominance of high-intensity low frequencies perceived beyond a certain loudness level. This implies that acoustically evoked sensations besides mere auditory ones may be sought. Two classes of sensations seem to be possible candidates here: *vibrotactile* (Verrillo, 1992; Levänen et al., 1998; Levänen and Hamdorf, 2001) and *vestibular* ones (Todd, 1993). The labyrinthine sensitivity to loud sound and vibration is well documented (Romand, 1992; Sheykholeslami and Kaga, 2002; Oertel and Young, 2004; Guinan, 2006; Phillips-Silver and Trainor, 2008), but it is still a matter of discussion whether stimuli, which are found in the sound environment of loud dance music, may evoke similar vestibular responses. There is already some physiological evidence for acoustic sensitivity of the vestibular system in the sense that, from an evolutionary point of view, the inner ear shows a division between the organs of balance (semi-circular canals and utricle) and those with an auditory function (saccule and lagena; Popper et al., 1982). At a later stage in evolution, the cochlea has come to replace the saccule as the primary organ of hearing, but there is still some evidence suggesting that the saccule has retained some acoustic function in higher vertebrates, such as amphibians, birds, and some mammals. It is the saccule, rather than the utricle or the semi-circular canals, which is maximally sensitive to sound (Todd et al., 2000, Todd, 2001; Colebatch, 2006; see also Emami et al., 2013). This same organ is also thought to mediate evoked myogenic responses to acoustic stimuli in humans, which are thus considered to have a vestibular rather than a cochlear origin. Such responses have been found, in fact, for music in dance clubs with sound levels above 90 dB(A) (sometimes approaching the intensity of 120 dB and even beyond) and in particular for frequencies between 200 and 400 Hz, which is close to those which are typically experienced in bars or dance clubs (100–300 Hz). The frequency distributions of dance club sounds—the rock and roll threshold—are thus well matched to the maximal sensitivity of the saccule. The question remains, however, why such acoustic saccular stimulation should be searched for? One of the possible explanations is the search for sensations of self-motion as obtained from swings, rocking chairs, and roller coasters, which are experienced for some as being equally pleasurable. As such, it is suggested that both these acoustically evoked saccular responses and vibrotactile sensation may be considered as possible sources of pleasure in loud music (Todd et al., 2000; Todd, 2001).

It thus seems that loud music is perceived primarily as a vibrational transduction of affect, rather than as a translation of meaning, with powerful lower frequencies that resonate with embodied movement. They seem to evidence a kind of sonic dominance, displaying a kind of “haecceity” or “this-ness” with a force of attack and sharpness of edge, as compared to the more tamed and domesticated mid-frequencies of what has been considered traditionally as music (Henriques, 2011, p. 38). As such, it is possible to conceive of “sound as force” in the context of bass culture rather than of “sound as text” (Goodman, 2009). One should be skeptical, however, about such metaphorical descriptions in terms of bass materialism, as conceived in the house-music culture of the 1990s. There is, in fact, still some controversy whether we actually “feel” the sub-bass frequencies in a physical sense rather than as a subjective experience, evoked by our reactions to the sounds. The lowest frequencies, in fact, fall outside of the rather narrow band between 2 and 5 kHz for which our ears are most sensitive. The bass-dominated sound, tuned into the sound waves below 100 Hz, moreover, has also not been primarily designed to produce “dystopian experiences of sonic domination” but was aimed rather at providing a distinctive sonic environment that “strove to envelop dancers in a shared physical experience of sound without punishing their ears” (Fink, 2018, p. 96). A deep, full-sounding sub-bass may be less fatiguing to the ear than highly amplified mid-frequencies, which stimulate the sensory systems more effectively due to the resonant properties of the ear, and at a higher rate, due to the frequency (Gleason, 2015).

As such, there seems to be misunderstanding about the so-called bass culture. Though it can be stated that low frequencies can impinge on the body in a haptic way, it should be considered that intermodal translation between the senses mostly involves an attenuation of intensity. This is the case, e.g., when trying to “feel” the music rather than merely “hearing” it. The ear, in fact, is one of our most delicate senses, which reacts to infinitesimally small portions of sound energy. This is not the case when the skin or another part of the body reacts to vibration. It should be noted, further, that air (the medium through which the sound waves are carried) and our bodies do not couple very well, due to a difference in acoustical impedance. This means that most of the long-wave energy of low-frequency sound bounces off the surface on our skin, leaving only a small fraction to impinge on the touch sensors in the epidermis. It is difficult to determine, further, how deep this effect goes, but for the head and the torso, this small surface displacement seems to engender the feeling of bass thump and punch inside the body (Fink, 2018). This is not the case, however, for the frequencies beyond the ultrasound border where small sound waves can intrude the interior of the body with high energy transmission (Altmann, 2001).

Most of the resonant frequencies of the human body are below the frequencies that loudspeakers can project, as most of the organs and viscera resonate most strongly around 5 Hz (Fink, 2018). This does not mean, however, that there is no feeling of vibration as the human body is sensitive to vibrations from 0.5 to 100 kHz, with the frequencies between 0.5 and 200 Hz as the most intrusive ones. This means that the felt vibrations from the lower frequencies may influence to some extent the haptic-tactile perception of low-frequency sounds as well as our subjective reaction to these sounds (Berglund et al., 1996).

This brings us to the phenomenon of liking or disliking loud music. What motivates listeners to listen at levels of discomfort, which are in the close vicinity of the threshold of pain? Is this an individual choice or should we consider also factors that go beyond conscious and deliberate control? A possible answer is to be found in social ecological models of behavior.

### Listening to Loud Sounds: Adaptation, Conditioning, and Acculturation

Listening to possible harmful sounds can be classified as health-risk behavior that accounts for both individual attitudes and beliefs and the impact of aspects of the social environment. The problem of possible harmful effects, however, is difficult to control as the personal rewards of loud music are quite immediate, whereas the harmful effects may become visible only after years (Blesser, 2007). The enjoyment of loud sound, moreover, appears to depend on a complex and powerful interaction of forces related to cultural, interpersonal, and intrapersonal factors. As such, it can be studied by applying the *Social Ecological Model* that considers four levels of influence: the intrapersonal level, the interpersonal level, the community level, and the policy level, all of them pointing toward an ecology of acceptance of high-level sound (McLeroy et al., 1988; see Richard et al., 2011 for an overview).

The *intrapersonal level* refers to the individual’s own thoughts and attitudes, reflecting personal preference for style and genre, as well as personality traits, which may influence the appreciation for loud sounds, such as sensation seeking behavior and a desire for rebelliousness (Arnett, 1994; Lozon and Bensimon, 2014). Loud music, in that case, is valued as providing intense stimulation and arousal. It has an exciting and arousing effect through stimulation of brainstem mechanisms, with connections to the reticular formation, which modulates our experience of sound and which may be expected to contribute to pleasurably heightened arousal (Juslin and Västfjäll, 2008). The *interpersonal level* refers to the direct influence of other associated people. It describes the influence of sound on interactions with others, reflecting the desire for group membership by adopting common styles and tastes (Bennett, 1999). The *community level* concerns the cultural influences on listener’s behavior. It refers to the accepted practices around loud music, such as the expectation of loudness from both nightclub staff and clubbers. Staff members, in particular, use loud music to market themselves in line with the conceptualization of a culture of loud sound and to influence their customers. Clubbers, as a matter of fact, seem to accept these loudness levels, even when they are experienced as being too loud. Levels of around 97 dBA Leq are not uncommon (Beach et al., 2013), mostly starting at a level of 85 dBA Leq but rising gradually through the course of the evening to reach this maximum level around midnight. The underlying mechanism is adaptation as the auditory system is highly adaptive to high-level sound, with physiological adaptation occurring at multiple sites in the cochlea (Fettiplace and Kim, 2014) and also in the cortex (Whitmire and Stanley, 2016). As such, sound levels are raising in order to meet what club managers consider to be the wishes of the customers who perceive loudness as a function of both the external level of sound and the degree of physiological adaptation. The *policy level*, finally, deals with the influence of legal requirements and aspects of government policy concerning noise levels in the workplace (McLeroy et al., 1988; see also Welch and Fremaux, 2017a,b). The Social Ecological Model, moreover, claims that influences toward good health should be present at each of the levels of the model in order to guarantee good health behavior.

**Figure 6:**
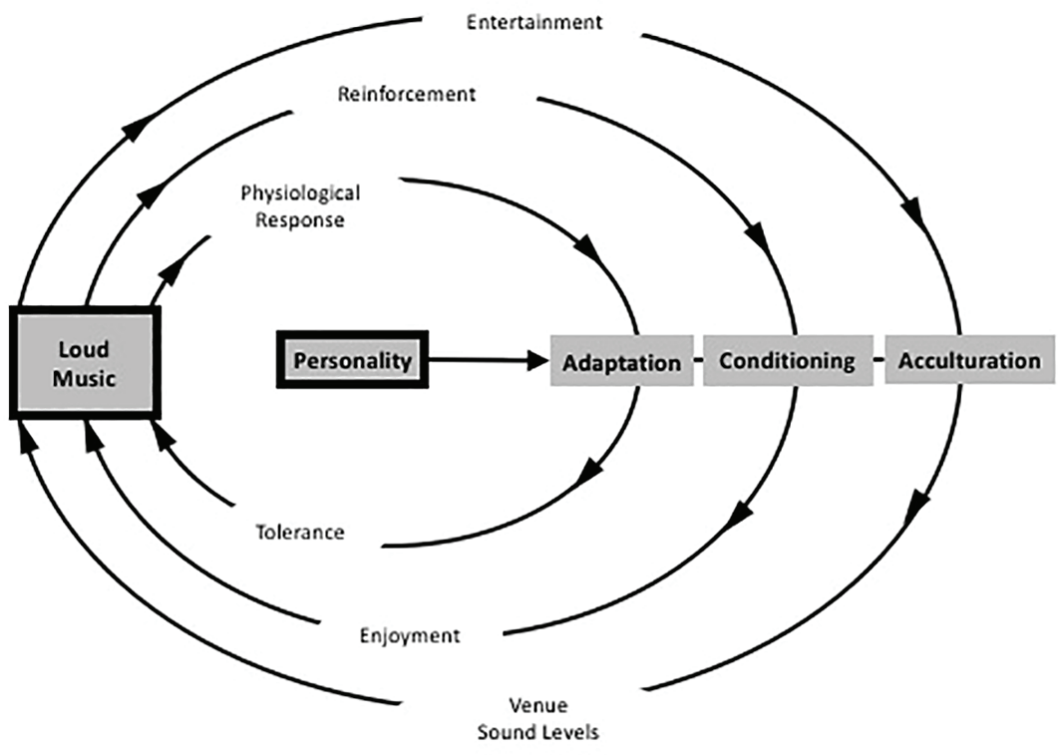
Schematic diagram of the CAALM Model diagram showing the three parallel cycles that may lead to people enjoying loud music, and the role of personality as a moderating factor. (Figure adapted and republished with permission of Thieme Publishers from Welch and Fremaux, 2017a).

A related theory has been proposed by Welch and Fremaux: the *CAALM model*, which is short for Conditioning, Adaptation, and Acculturation to Loud Music (Welch and Fremaux, 2017a,b). It is based on three processes: (1) an initial adaptation that should enable listeners to overcome the experienced discomfort that is associated with loud music; (2) a classically conditioned response that repeatedly pairs levels of loudness with perceived benefits of loudness such as masking of other unwanted sound, social benefits, arousal, excitement, and other associated benefits such as dancing, fun, friends, alcohol, or other substances, and (3) an acculturation process wherein large groups of listeners start to perceive loud music as the norm and as the common association of fun (see Figure 6).

Underlying the model are several positive features of loud sound that contribute to the conditioning effect: (1) loud music masks unwanted sounds, (2) it enables more and greater socialization (cf. Cusick, 2006), (3) it provides opportunities for intimacy in crowded space, (4) it masks unpleasant thoughts, (5) it is arousing, and (6) it emphasizes personal identity, especially personal toughness and masculinity. The latter reflects culturally accepted norms of masculinity as being associated with activity and danger and may represent the neural interaction where a natural fear response would occur to the loud sound and then the person is able to exert control over that response, thus generating a feeling of strength (Welch and Fremaux, 2017b).

## Conclusions

The hearing systems in the ancestral lineage that led to *H. sapiens* have evolved in response to stable environmental conditions as far as the physics of sound is concerned (Lewis and Fay, 2004). As a result, our neural mechanisms for sound detection are characterized by many traits shared with other species. Therefore, the use of noise and loud sounds in music affects our hearing system independently of our music-specific interpretation of sound. After all, music as a human-specific form of communication has evolved only recently among hominins (Mithen, 2006). Bearing this in mind, it is reasonable to suppose that the role of infrasound and lower-frequency sounds in music is not primary as far as the recognition of musical structure is considered. This is especially true in the process of pitch structure perception as the precision of spectral analysis that occurs in the auditory system seems to be indispensable for pitch experience. Nevertheless, music as a vibrational energy is also a source of many extra-structural features that cannot be underestimated as the parts of our sensation of music. From this perspective, the role of the vestibular and tactile systems being involved in the process of infrasound and lower-frequency sound detection should be treated as a part of multimodal music experience. It is possible that thanks to the fast-developing sound technology, this part of music experience will become more important.

Care should be taken, further, with respect to broadly accepted ways of listening to sound levels above the threshold of discomfort. Liking such overstimulation is likely to spiral into patterns of addiction. The concept of *maladaptive listening* (Miranda and Claes, 2009; Garrido and Schubert, 2013) can be used in this context. Addiction, in fact, has been traditionally seen as being based around the concept of pathological usurpation of neural processes that normally serve reward-related learning. It can be considered as a maladaptive habit formation that involves the dopaminergic circuits of the brain, such as the nervus accumbens, the ventral tegmental area, the dorsal striatum, and the prefrontal cortex (Hyman et al., 2006). The case of music is quite interesting in this regard because of possible couplings with vestibular self-stimulation—also called the “dance habit,” which plays an important role in beat (Todd and Lee, 2015) and meter induction (Trainor et al., 2009; Trainor and Unrau, 2009) and allows a rapid reward-based selection of self-motion of the body in the sensory-motor circuits of the supplementary motor area (SMA) and the cingulate motor area (CMA) of the brain (Todd and Lee, 2015).

Listening to loud music, finally, seems to activate primitive mechanisms of experience, evoking to some extent an amodal kind of perception and surpassing to some extent boundaries between sensory modalities. It can be hypothesized that this is a return to the oceanic feeling or state, as suggested by Freud (Saarinen, 2012), or a desire to be surrounded by a cocoon of sound, as suggested by the rock and roll threshold with its excessive vibrotactile and haptic stimulation. People may find unusual stimulation of all kinds pleasurable, even when flirting with the threshold of pain.

## Author Contributions

The first draft of this manuscript was prepared by MR. The final text was written jointly by MR, DW and PP.

### Conflict of Interest Statement

The authors declare that the research was conducted in the absence of any commercial or financial relationships that could be construed as a potential conflict of interest.
